# Dynamic capabilities and employment during COVID-19: The moderating effect of government support

**DOI:** 10.1177/02662426231173352

**Published:** 2023-05-28

**Authors:** Karolis Matikonis, Byron Graham

**Affiliations:** Queen’s University Belfast, UK; Queen’s University Belfast, UK

**Keywords:** dynamic capabilities, employment, government support, COVID-19

## Abstract

Although small and medium-sized enterprises (SMEs) were particularly hard hit by the coronavirus disease 2019 (COVID-19) pandemic, some performed relatively well, maintaining or increasing employment growth. We study these differences in performance through the lens of dynamic capabilities (DCs) theory, which we extend by incorporating the moderating effect of government support during COVID-19. We analyse responses from 1421 UK SMEs and find that government support and DCs positively impact employment. We also show that government support moderates the link between DCs and employment with a negative effect after the first COVID-19 lockdown. The findings highlight the role of government policy intervention in discouraging SMEs from effectively exercising DCs during a crisis and the general importance of DCs and government support in enabling SMEs to cope with shocks.

## Introduction

The coronavirus disease 2019 (COVID-19) pandemic had a substantial impact on businesses across most industries, with many firms suffering from decreased revenue, employee layoffs and closures (Albonico et al., 2020; Bartik et al., 2020a; Donthu and Gustafsson, 2020; Office for National Statistics, 2022a). Small and medium-sized enterprises (SMEs) were particularly negatively affected (Liguori and Pittz, 2020) due to their smaller size, lower cash holdings (Calabrese et al., 2022; Cowling et al., 2020; Eggers, 2020) and a relatively higher proportion of SMEs in industries impacted by COVID-19 constraints, such as social distancing in the service sector (Belitski et al., 2022).

To mitigate the negative impact of COVID-19, most governments introduced support mechanisms, including loan and furlough schemes, to help reduce redundancies and risk of failure (Calabrese et al., 2022; Khlystova et al., 2022). In the United Kingdom, these assistance schemes were extensive, with estimated costs of over £147 billion (National Audit Office, 2022). The initial findings indicate that they helped firms during the pandemic by contributing to the reduction in failure rates and employee layoffs (Belghitar et al., 2022; Cowling et al., 2022).

Although the pandemic has had an overall negative impact on the economy and, in particular, on SMEs (Belitski et al., 2022), some firms have performed relatively well, increasing sales and employee growth (Dannenberg et al., 2020; Dhawan, 2020; Khlystova et al., 2022; Rowan and Galanakis, 2020). However, the mechanisms behind these performance differences are not well understood. One strand of research (Clampit et al., 2022; Dejardin et al., 2022; Dovbischuk, 2022; Marco-Lajara et al., 2021) has highlighted the importance of dynamic capabilities (DCs) in enabling firms to cope with shocks such as the COVID-19 pandemic. This research has identified a positive relationship between DCs and firm performance. However, past studies have not considered the moderating role of government support mechanisms in shaping firms’ ability to exercise their DCs to cope with the challenges of the COVID-19 pandemic.

It is not clear from the extant literature how government support initiatives interact with DCs. Although government support schemes, such as loan guarantees, helped to protect businesses and jobs during the pandemic (Cowling et al., 2022), some studies have highlighted the unintended negative consequences of expansive assistance programmes, especially in the longer term. These included a rising proportion of non-viable firms that are alive because of assistance negatively impacting employment (Acharya et al., 2020, 2022; Caballero et al., 2008; McGowan et al., 2018), among other indicators. Although past research provided valuable insight into the role of DCs and government support, it has not examined the potential for unintended consequences arising from COVID-19 government support, particularly the interaction between support and DCs. Examining this is important, as it could enable us to understand the role of government policy intervention in discouraging SMEs from effectively exercising DCs and consequently providing an evidence base for better policymaking.

This study aims to contribute to these gaps in the literature by studying the impact of DCs on employment during the COVID-19 pandemic and the moderating role of government support. We question whether the unprecedented government support could have unintended effects on DCs. To this end, we empirically test hypotheses, informed by DCs theory, with a representative sample of 1421 micro, small and medium-sized enterprises to understand how DCs affect changes in employment during the COVID-19 pandemic and whether the government support moderates this link. The results of our study highlight the positive impact of DCs and government support on employment. Most importantly, we show that the moderation effect becomes negative for employment growth aspirations after the first lockdown, signalling the adverse longer-term impact of government support on DCs. From a practical perspective, this study thus calls attention to the role of government policy intervention in enabling SMEs to exercise DCs during a crisis and the general importance of DCs and government support in enabling SMEs to cope with shocks.

In this article, we contribute to the literature on the impact of COVID-19 on SMEs, their resources (Brown et al., 2020; Cowling et al., 2020; Martinez Dy and Jayawarna, 2020; Roper and Turner, 2020), business models (Guckenbiehl and Corral de Zubielqui, 2022), government support (Cowling et al., 2022; Yue and Cowling, 2021) and employment (Brown and Cowling, 2021; Cowling et al., 2022). We draw on theory and empirical results from past studies in these areas to develop our hypotheses linking DCs, government support and employment. Our extensive micro-level data further supplement and extend more aggregate-level studies on employment dynamics (Brown and Cowling, 2021; Cowling et al., 2022) and enable capturing a broader range of support mechanisms than past studies (Cowling et al., 2022; Yue and Cowling, 2021). We also contribute to the DCs literature, building on the work of Clampit et al. (2022), by assessing whether their findings also apply to employment dynamics and whether this is moderated by government support.

The remainder of the article proceeds as follows. We commence with an overview of employment dynamics and government assistance during the COVID-19 pandemic in the United Kingdom, which helps with the positioning and design of our study. We then review the existing literature to develop our hypotheses linking DCs, government support and employment dynamics. This is followed by the description of our data and measurements and reporting of results from ordinal regressions. The paper then discusses the theoretical and practical implications arising from the results, before presenting conclusions and limitations.

## Background

The COVID-19 pandemic placed a substantial burden on businesses, in particular during the unprecedented first lockdowns. In the United Kingdom, the government announced the first national lockdown on 23 March 2020, which is less than 2 months after the first two cases of COVID-19 were discovered in the United Kingdom. The government ordered people to stay home and all non-essential businesses to close or switch to remote working. For many, this overnight switch from onsite to remote working during the first lockdown resulted in increased anxiety and depression (Dettmann et al., 2022), uncertainty (Kim and Asbury, 2020) and decreased productivity (Rietveld et al., 2022). Those in essential sectors allowed to operate were exposed to various restrictions^1^ and a constantly changing marketplace, triggering unexpected outcomes across sectors (Panzone et al., 2021). The restrictions were slowly relaxed starting in May, with most of them lifted in July. Non-essential businesses were permitted to open, and gatherings of up to 30 people were again allowed. From mid-September, the restrictions again started to be tightened with the imposition of further limitations on gatherings and opening times of restaurants and bars.

**Figure 1.**
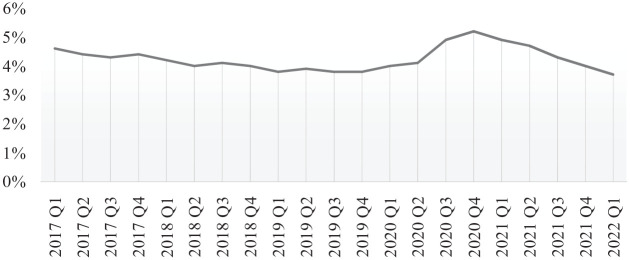
Unemployment rate (aged 16 and over). Writers’ illustration based on data from Office for National Statistics (2022b).

Although other COVID-19 phases followed similar patterns, with restrictions to some extent re-imposed through two further national lockdowns (November 2020 and January to March 2021), these were less unexpected for businesses, shorter, better managed and generally less severe than during the first lockdown (Barber et al., 2022; Brown and Kirk-Wade, 2021). This is also evident when looking at the employment changes in Figure 1. Unemployment rose sharply, peaking at 5.2% in the second quarter of 2020, reaching some of the highest levels since the recovery after the great recession (Office for National Statistics, 2022b). As restrictions were progressively lifted, unemployment swiftly decreased to pre-pandemic levels, with employment figures for the first quarter of 2022 surpassing the fourth quarter of 2019, as illustrated in Figure 1.

## Hypotheses development

**Figure 2.**
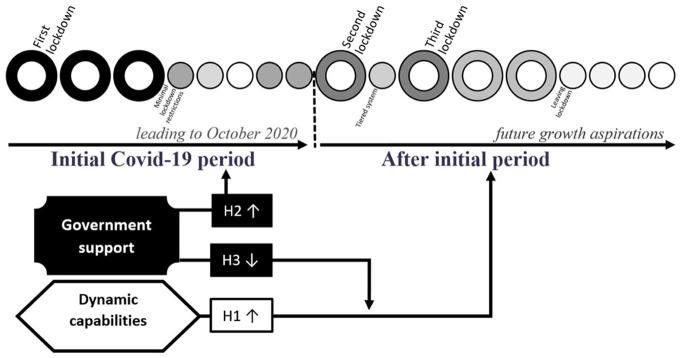
Theoretical model. Author’s illustration. Arrows represent the changes in employment, in which ↑ refers to employment growth and ↓ to reduction. Circles relate to the months commencing with March 2020. The shaded colours in the coronavirus disease 2019 (COVID-19) timeline refer to the severity of restrictions, with black indicating severe restrictions and white almost no restrictions. The COVID-19 timeline is based on Barber et al. (2022) and Brown and Kirk-Wade (2021).

The hypotheses for the study, developed in this section, focus on the interplay between the receipt of government support and firm-level DCs during COVID-19. We thus hypothesise on two core research questions. The first focuses on whether DCs have an impact upon SME employment. The second is twofold and covers the impact of government support on SME employment and its role in moderating the relationship between DCs and SME employment. Figure 2 presents the overall conceptual framework for the study, illustrating the relationship between the three hypotheses.

In developing the hypotheses, we consider two timeframes. The first reflects the shock of the first lockdown during the COVID-19 pandemic by capturing employment change over the 12 months prior to survey completion, with survey data collection taking place in September and October 2020. We chose this timeframe because it covers the period incorporating the first lockdown until just before the second lockdown and thus allows enough time for the initial impacts of the COVID-19 shock on employment to be felt by SMEs. It includes imposing and lifting one round of restrictions (Brown and Kirk-Wade, 2021) and the introduction of most government support mechanisms (Barber et al., 2022), allowing for their initial impact also to be measured. During this timeframe, firms also began to adapt by moving online and introducing improved remote working practices and technologies (Martin et al., 2022). The second period captures the employment growth aspirations over the 12 months following survey completion.

### Do DCs affect SME employment during COVID-19?

To better explain firm responses to the turbulence resulting from unprecedented changes in demand and the need for different services during the COVID-19 pandemic, we adopt DCs theory considered one of the most significant developments in the strategic management literature (Helfat and Peteraf, 2009). DCs extend the resource-based view that sees resources as key to gaining and sustaining competitive advantage over time by incorporating dynamic settings (Teece and Pisano, 1994). The acceptance of DC theory resulted in numerous, sometimes conflicting extensions, examined through various methodologies. Some (Arend, 2014; Schilke et al., 2018) argued that the variety of theories places DCs at a disadvantage, whilst others (Helfat and Peteraf, 2009) reasoned this to be unavoidable and even welcomed, given the complexity of organisations.

Although many different conceptualisations of organisational capabilities have been developed, the broad consensus is that ordinary (or zero-level) capabilities contrast with DCs. The former are directed towards maintaining outputs by using and coordinating tangible and intangible resources, enabling an organisation to ‘make a living’ (Winter, 2000, 2003). DCs differ from ordinary capabilities in their ability to routinely alter an organisation’s way of making a living by focusing on ‘the capacity of an organization to purposefully create, extend, or modify its resource base’ (Helfat et al., 2007: 4), which is found to be essential during external shocks, such as COVID-19 (Clampit et al., 2022). The complexities of DCs are highlighted by Eisenhardt and Martin (2000), who point to equifinality (have different starting points and follow unique paths), substitutability (differ in form and detail) and fungibility (effective across a range of industries) in their efforts to define DCs. This is further amplified once we consider that the differentiating line could be blurry even among these two widely applied concepts (Helfat and Winter, 2011).

Once we delve deeper into the DC theory, the concepts become even less definite. This is evident once we attempt to differentiate the change that is caused by DCs from that caused by non-routine ‘ad hoc problem solving’ (Winter, 2003), which fits well in the COVID-19 context. Unprepared SMEs that previously were not routinely seeking change start to innovate due to the necessity imposed by COVID-19. It is, however, arguable to what extent these substantial changes in operation could be considered purely ad hoc problem solving. It is likely that these changes, to some extent, resulted from the previous experience through which DCs ordinarily develop (Bingham et al., 2015), especially for those SMEs that swiftly started to change during the first COVID-19 lockdown. They were also often required to engage with information technology, directing more towards improvisational capabilities introduced by Pavlou and Sawy (2010). Even when introducing the concept of ad hoc problem solving, Winter (2003: 993) acknowledged that ‘some of the pattern may be learned and contribute positively to effectiveness. . . it is not necessary that the pure forms [of ad hoc problem solving] exist in the world.’

Thus, it seems naïve to strictly link the multifaceted phenomenon of SME change with a particular notion from the DC theory. In our quantification of DCs, we join numerous scholars (Arend, 2014; Clampit et al., 2022; Kump et al., 2019; Pavlou and Sawy, 2010), who combine various measures to estimate specific DCs that are relevant for a particular study and/or setting to capture discrete subchannels of interest. This enables us to more directly measure the effects on SMEs that exploited DCs during the COVID-19 shock without precisely defining their historical routineness, equifinality, substitutability or fungibility.

There is also a lack of empirical consensus on the mechanisms through which DCs operate (Barreto, 2010) and how they should affect the performance of firms (Baia et al., 2020; Wilden et al., 2016; Zahra et al., 2006). Having said that, the initial rationale offered by Teece et al. (1997) and many subsequent studies argued that DCs are expected to positively impact performance, especially when disruption is high, because of their ability to add value when settings are dynamic (Helfat and Peteraf, 2009; Schilke, 2014; Teece et al., 1997). The explanation of mechanisms through which this is achieved varied but was often linked to the ability to adjust how resources are configured under changing environments in which firms operate (Jarzabkowski and Wilson, 2006; Teece et al., 1997). The extent of the effect is likely to depend on the nature of the DC and whether the repeatable function of that particular DC is appropriate for that particular setting (Helfat et al., 2007).

For instance, SMEs that have an existing DC of adopting various business models could utilise it in the context of COVID-19 by promptly adapting their business models to overcome the constraints imposed by the pandemic. There are many avenues through which this can be achieved, such as through building resilience (Ali et al., 2022) or business model innovation (Breier et al., 2021), by engaging in business model experimentation (Björklund et al., 2020) and, in this way, benefiting from the crises (Seetharaman, 2020). We already have many examples where COVID-19 enabled entrepreneurs to gain advantages from changes to the business environment (Davidsson et al., 2021; Guckenbiehl and Corral de Zubielqui, 2022; Klyver and Nielsen, 2021), and for them, COVID-19 triggered growth (Donthu and Gustafsson, 2020), especially by creating opportunities in online food retail (Dannenberg et al., 2020), agri-food (Rowan and Galanakis, 2020) and education (Dhawan, 2020).

Similarly, the limited number of empirical studies examining how DCs affect SMEs during COVID-19 direct towards positive impact on performance. Clampit et al. (2022) theorised that during the COVID-19 pandemic, the ability to make prompt decisions on how to operate is vital, and the need to do so is much stronger because of the increased risks of closure. They tested this with data from 338 US SMEs and found that DCs used during lockdowns had a positive association with operational levels and turnover. Similar findings were reported by Dejardin et al. (2022), who examined data from 209 Portuguese SMEs. We extend these studies to employment growth and further suggest that SMEs with greater operation levels and turnover (Clampit et al., 2022; Dejardin et al., 2022) resulting from DCs utilised during the first lockdown had more confidence that they could adapt and grow despite the impact of the pandemic. It is, however, worth noting that organisational growth is a multifaceted phenomenon, and employment growth is determined by largely distinct factors that are different from other indicators (Janssen, 2009). In some cases, these were found to exist very independently. For instance, although innovation was positively associated with employment growth, primarily negative associations were found between innovation and the growth in turnover, productivity and profit margins (Freel and Robson, 2004).

Another factor to consider is the potential uneven pace of adaptation to the COVID-19 pandemic, with some SMEs adopting a wait-and-see approach. SMEs may be unsure what to do and thus decide to wait before taking any action, altering the timeframe and extent of the DCs’ effect on performance (Pavlou and Sawy, 2010; Wilhelm et al., 2015) and possibly employment growth. Business model innovation in a digital context, which is likely to be prominent during COVID-19, has been found to centre around experimentation (McGrath, 2010), adaptation and learning (Sosna et al., 2010). Andersen et al. (2022) extend these ideas to the perspective of SMEs. They established four key activities: ‘(1) scanning the business environment, (2) conveying a sense of urgency, (3) experimenting with digital innovation and (4) shifting decision-making from intuition to data’ (Andersen et al., 2022:30–31). Arguably, SMEs with higher levels of DCs would focus on longer-term performance (Teece, 2007), including employment growth. They would prioritise urgency in favour of a wait-and-see approach but not necessarily at the expense of scanning the business environment, experimentation and learning from the data (Andersen et al., 2022), indicating less uniform short-term effects of DCs on employment.

Furthermore, the pace of adaptation may also be a product of varying restrictions, further contributing to the prospect of a somewhat uneven or delayed effect of DCs on employment change. SMEs had little input in the design of lockdowns, including the severity and extent of restrictions and subsequent lifting and re-imposition of them (Brown and Kirk-Wade, 2021), which could potentially impact the speed of adaptation to the pandemic. For instance, SMEs that were not allowed to operate in their high street premises would be more encouraged to utilise their DCs to shift to an alternative marketplace, with findings pointing to even ethnic minority restaurant owners who were previously reluctant to innovate but highly affected by restrictions, promptly embracing innovation (Korede et al., 2023), and thus these SMEs could potentially have more time to learn, experiment and adjust their employment. Whilst SMEs that were less affected by restrictions, for instance, those classified as essential businesses (Fairlie and Fossen, 2022) may implement changes later in the pandemic. Although they would benefit from more time for scanning the business environment and data collection, they would have less time to learn from their own experience and adjust resources, including employment.

To mitigate this concern of non-uniform short-term effects resulting from the uneven pace of adaptation, we join numerous scholars (Darnihamedani and Terjesen, 2022; Davis and Shaver, 2012; Efendic et al., 2015) and shift our focus to employment growth aspirations over the subsequent 12 months from the end of the first lockdown in our efforts to capture DC effects. Growth aspirations have long been known to predict actual future growth (Wiklund and Shepherd, 2003). Therefore, our first hypothesis tests whether companies with higher levels of DCs had more confidence that they could adapt and grow in the face of the COVID-19 pandemic:

*Hypothesis 1.* Dynamic capabilities positively contribute to SME employment after the first lockdown of the COVID-19 pandemic.

### Does government support affect SME employment during COVID-19?

To address our second research question, which centres on government support, we commence by discussing the main COVID-19 support instruments and considering their shorter-term effects on employment. A large proportion of the COVID-19 assistance was through grants that are direct payments from the central government and do not need to be repaid. They were primarily designed to compensate businesses forced to close or significantly adjust operations due to restrictions. In terms of targeting of the COVID-19 assistance, the UK government specifically focused its efforts on supporting, protecting and creating jobs. For instance, during the summer Economic Update on 8 July 2020, the then Chancellor of the Exchequer, Rishi Sunak, announced the ‘Plan for Jobs’, introducing various support programmes to mitigate the impact of the pandemic (HM Treasury, 2020a) and justified the introduction of the *Coronavirus Job Retention Scheme (CJRS)*, with ‘I have a responsibility to make sure we protect, as far as possible, people’s jobs and incomes’ (HM Treasury, 2020b). *CJRS* is the costliest instrument, effective between March 2020 and September 2021. The scheme helped employers pay their workers’ wages if they were unable to work during the COVID-19 pandemic. Employers were able to claim grants, covering up to 80% of an employee’s usual wages up to a total of £2500 each month. Twenty-one percent of employing businesses participated in the scheme, with 1.16 million jobs on furlough and an estimated cost of £70 billion at the end of the scheme (Powell et al., 2021). Broadly similar but less extensive (Yue and Cowling, 2021) support was provided for the self-employed via the *Self-Employment Income Support Scheme (SEISS)* with estimated costs of £28 billion, supporting 2.9 million individuals (National Audit Office, 2022).

Other COVID-19 support instruments were less directly related to employment. The most prominent tax cut was *Business Rates Holiday (BRH)*, with an estimated cost of £17 billion. Relief from commercial property taxation was introduced for the majority of businesses. Those that operate in retail, hospitality and leisure businesses, and nurseries, had their business rates waived for the 2020/21 tax year. The support was later extended until 30 June 2021 when rates were reintroduced at a two-thirds discount. Amongst other support measures, the government also offered *value added tax deferral (VATD)* and *His Majesty’s Revenue & Customs (HMRC) Time to Pay (TtP)*. The latter deferral applied from 20 March to 30 June 2020 and was offered automatically, so businesses were not required to make an application. A total of £33.5 billion was deferred over the period. Whilst the existing TtP scheme was extended by scrapping the usual 3.5% annual interest on deferred payments and offering more extended periods to settle the tax bills.

Although other COVID-19 support instruments, such as *BRH*, are less directly related to employment, they should also trigger positive effects, similarly to other tax incentives (Liu et al., 2019; Sterlacchini and Venturini, 2019; Venâncio et al., 2022). We typically assume that public resources spent on support instruments will return to the economy through overall regional economic development by increasing competitiveness and creating employment (Cowling and Dvouletý, 2022; Kersten et al., 2017). The support should relieve businesses of cashflow problems, amplified by the shock in demand or operations and, in this way, enable them to sustain employment during an external shock (Biggs et al., 2012; Khan and Sayem, 2013), such as the COVID-19 pandemic. Furthermore, the support also has the potential to enable SMEs to focus on taking advantage of opportunities for growth that arise during the pandemic, of which we have numerous examples (Dannenberg et al., 2020; Dhawan, 2020; Khlystova et al., 2022; Rowan and Galanakis, 2020).

This reasoning is broadly supported by the existing empirical findings on the effectiveness of government assistance during COVID-19 that consistently point to a largely positive impact on employment during the initial stages of the pandemic. Gourinchas et al. (2020) found that there would be a substantial increase in the failure rate of SMEs during COVID-19 if there were no government assistance. For the UK, Cowling et al. (2022) measured the effects of the Bounce Back Loan, which was introduced by the UK government to guarantee loans up to £50,000. They estimated losses of £7–£12 billion, but they argued that the schemes protected 118,639 businesses and 1,117,849 jobs. In the US, the largest assistance programme during COVID-19 was the Paycheck Protection Program (PPP), providing loans for SMEs through private sector lenders. Although the design of the scheme was criticised for its inherently different objectives: employment protection for the government and profits for banks (Joaquim and Netto, 2021), the empirical findings suggest that PPPs saved many jobs among micro firms (Doniger and Kay, 2021) and increased their prospects of survival (Bartik et al., 2020b).

This encourages us to reason with the second hypothesis that government assistance is likely to positively contribute to employment in the initial stages of the COVID-19 pandemic directly through furlough schemes (*CJRS* and *SEISS*) and also through less direct support (*VATD, TtP* and *BRH*) by promptly closing the resource gaps that are particularly pronounced among small businesses (Calabrese et al., 2022; Cowling et al., 2020; Cowling and Dvouletý, 2022; Eggers, 2020):

*Hypothesis 2.* Government support positively contributes to SME employment during the first lockdown of the COVID-19 pandemic.

### Does the COVID-19 government support moderate the effects of DCs on employment?

The longer-term implications of COVID-19 government support on employment are less straightforward. Firstly, the benefit from the support could depend on the existing DCs amongst SMEs. Government support may be less effective for SMEs that already have relevant DCs that are related to resilience (Ali et al., 2022), developed through business model innovation (Breier et al., 2021), or experimentation (Björklund et al., 2020), for example. They could potentially swiftly utilise these DCs in the context of COVID-19 and achieve a competitive advantage without input from the government. This is, to some extent, evident by looking at *BRH.* Some *BRH* recipients performed exceptionally well during the COVID-19 pandemic, with several voluntarily paying back the relief with estimates of over £2 billion returned to the Exchequer (Office for Budget Responsibility, 2021: 202).

On the other hand, the availability of expansive multibillion-pound support packages, with an estimated value of £147 billion,^2^ and early announcements of further support schemes to aid with the recovery from the COVID-19 pandemic may also divert businesses from drawing on their relevant DCs and further developing relevant experience through adapting to the new market conditions. Initial evidence from PPPs indicates that businesses often used COVID-19 loans to make non-payroll fixed payments and build up savings buffers (Granja et al., 2022), but DCs are known to be developed by learning through experience (Bingham et al., 2015). The increased reliance on temporary cash influx from the government could potentially negatively impact SMEs by losing the long-term competitive advantage to firms that further developed their DC base through experience during the COVID-19 pandemic, of which we already have numerous examples (Davidsson et al., 2021; Guckenbiehl and Corral de Zubielqui, 2022; Klyver and Nielsen, 2021). Furthermore, some instruments adopted during the COVID-19 pandemic were already shown not to have positive longer-term outcomes. For instance, Small Business Rate Relief, which shares underlying mechanisms with *BRH* and related loans targeted at small businesses that were applied during COVID-19 (Matikonis, 2020), was already shown not to stimulate longer-term employment (Gobey and Matikonis, 2021).

One stream of the literature goes even further and suggests that enabling distressed firms to stay afloat has negative, longer-term consequences. For instance, McGowan et al. (2018) found a rising proportion of firms that were non-viable but kept alive because of the instruments made available during the financial crisis, including SME support policy initiatives. A large proportion of these firms were found to negatively affect other firms operating in the same industry-country pairs (Acharya et al., 2020; McGowan et al., 2018). They tend to be associated with lower churn, higher markups and, most importantly, the misallocation of labour and capital that results in lower productivity, investment and added-value (Acharya et al., 2020).

Building on Acharya et al.’s (2020) mechanisms that explain this phenomenon through a lack of adjustment in the aggregate production capacity after a negative demand shock, we could also attribute this to the lack of DCs. SMEs lacking in ‘the capacity . . . to purposefully create, extend, or modify its resource base’ (Helfat et al., 2007: 4) take a share of those that have DCs, contributing to the lack of overall effectiveness of DCs and subsequent lower employment growth. Other businesses operating in industries exposed to a large number of these non-viable firms were also found to experience lower employment growth (Acharya et al., 2020, 2022; Caballero et al., 2008; McGowan et al., 2018), among other indicators.

This could be amplified by the hastily introduced support instruments without considering their longer-term unintended consequences. For instance, *BRH* and related loans available for businesses occupying less expensive premises could be distortionary. The latter loans are argued (Matikonis, 2020) to be mistargeted, because their availability criterion is solely based on the size and value of the premises they occupy. The former is directed to larger businesses because the smaller businesses were already eligible for the discount through Small Business Rates Relief (Matikonis, 2022). The poorly targeted support could also result in harmful longer-term consequences by randomly equipping some competing firms, irrespectively of their DCs, with extra resources and, in this way, providing a competitive edge over their competitors that potentially already have established DCs.

There are many avenues through which DCs could become inferior or less effective because of government support, especially in the longer term. For instance, the government could unintentionally encourage a shift towards exchanging productivity-enhancing DCs with political management capabilities through the increasingly dynamic political environment with changing support mechanisms. To stay competitive in this environment, firms are required to establish dynamic political management capabilities, as explained by Oliver and Holzinger (2008), to successfully acquire optimal and frequently changing assistance and exploit government policies. The impact of the lack of this capability among small firms is highlighted by Balyuk et al. (2021), who find that small firms were less likely to get PPP loans in the early stage of the programme, but this was not the case for the firms that took loans previously. The effectiveness of political management capabilities could gradually decrease, as they are more likely to be targeted at only immediate generic subsidies or tax reductions that are not likely to be extended after COVID-19.

These non-viable firms are likely to be covered by the COVID-19 assistance programme, owing to the uniform support with broad eligibility criteria enabling most companies to claim COVID-19 support. The findings indicate that before the COVID-19 pandemic, supported firms were more likely to exhibit low productivity (Morikawa, 2021) and have lower credit scores (Hoshi et al., 2023), pointing towards SMEs with inferior or less effective DCs. Although during the COVID-19 pandemic, it is effective to swiftly supply cashflow-constrained but healthy firms, the generous support initiatives also could have sustained and created new, less viable firms that could potentially, as Hoshi et al. (2023: 15) argues, ‘transform the temporary shock due to COVID-19 into a permanent shock by distorting the liquidity supply toward inefficient firms.’ This suggests that the government could be spending considerable resources to enable possibly non-viable firms with no intention or confidence to grow to stay alive, negatively affecting other firms operating in the same industries through the misallocation of resources. This leads us to propose an adverse moderating effect in our third hypothesis:

*Hypothesis 3.* Government support negatively moderates the link between dynamic capabilities and SME employment after the first lockdown of the COVID-19 pandemic.

## Data

To test our hypotheses, we use data from the Longitudinal Small Business Survey (LSBS), commissioned by the Department for Business, Energy and Industrial Strategy. LSBS is a large-scale survey of small business owners and managers from across each of the four UK regions (England, Scotland, Wales and Northern Ireland). SMEs are defined as businesses with less than 250 employees. The data was collected via a telephone survey, with the sample devised by stratifying based on region, sector (1 digit in the Standard Industrial Classification framework) and size of businesses. The Inter Departmental Business Register was used as the sample source for registered businesses and Experian for those unregistered with no employees. The data is cross-sectional because we limit the data collection to one period between September and October 2020.^3^ The final sample size is 1421 for the models focusing on employment changes between the survey date and 12 months prior, and 1294 responses for the models focusing on growth aspirations between the survey date and the following 12 months.

## Measures and methods

### Dependent variables

Two ordinal dependent variables are constructed for the analysis, focusing on two different time periods. The first dependent variable focuses on employment changes between the survey date and 12 months prior, thus covering the time period between September/October 2019 and September/October 2020. This measure is based on responses to the survey question: ‘How many employees did the business have on the payroll 12 months ago across all UK sites (still excluding owners and partners)?’ with possible choices of *more than currently, the same, fewer* and *don’t know*. Those that responded *don’t know* were excluded from the analysis, and others were recoded into the three corresponding categories: *decrease in employment, the same* and *increase in employment*.

The second dependent variable focuses on the expected change in employment levels between the date of the survey and 12 months after the survey date, thus covering the time period from September/October 2020 until September/October 2021. This variable is constructed based on responses to the survey questions: (1) *Approximately how many employees are currently on your payroll in the United Kingdom, excluding owners and partners, across all sites?* (2) *How many employees do you expect the business to have on the payroll in the United Kingdom in 12* *months’ time (excluding owners and partners)?* The variable for inclusion in the regression analysis is constructed by dividing the expected employment in 12 months by the current level of employment. This is then collapsed into three categories, providing an ordinal variable which can take the following values: *decrease in employment, the same* and *increase in employment*. A decrease in employment is defined as firms whose employment change is less than 1, and an increase is defined as an employment change of greater than one. Firms with a score of 0 do not change employment levels. Measures of growth aspirations are commonly applied in the wider entrepreneurship literature (Darnihamedani and Terjesen, 2022; Davis and Shaver, 2012; Efendic et al., 2015).

### Independent variables

The focal independent variables for the study include DCs and the government support mechanisms received by the firm. In our quantification of DCs, we join numerous scholars (Arend, 2014; Clampit et al., 2022; Kump et al., 2019; Pavlou and Sawy, 2010), who combine various measures to estimate specific DCs that are relevant for a particular study and/or setting to capture discrete subchannels of interest. This enables us to capture DCs related to COVID-19. This approach to measuring DCs is common in the wider literature because of the highly multidimensional nature of the construct and the lack of consensus around the definition and measurement of DCs (Arend and Bromiley, 2009; Døving and Gooderham, 2008).

DCs in our specification range from 0 to 5 and capture the number of COVID-19 strategies adopted. We start from 0 and add 1 for each instance when DCs are demonstrated. This consists of a business reporting whether there was: (1) a change in services/products; (2) a change in processes/ways of working to mitigate the impacts of the pandemic and any associated trading restrictions; (3) an increase in operations during the COVID-19 pandemic; (4) whether the business increased and/or introduced online/virtual training and (5) whether they facilitated a change in the methods of selling and/or started selling online as a result of COVID-19.

It is worth noting that the available variables limited our specification. We extensively consulted with literature on single and multi-item measures commonly applied to quantify DCs, as in Døving and Gooderham’s (2008). We also draw on the work of Clampit et al. (2022) when developing our measure of DCs, who constructed their measure of DCs by combining three items focusing on the development of capabilities during COVID-19. To gauge the construct validity, we also extensively discussed the measurement with subject experts.

The measure of government support captures the number of government assistance programmes received by the SME. It can take any value between 0 and 5, with 0 indicating the business received no support and 5 indicating the business received support from five mechanisms. The survey asked respondents to report whether they had received assistance from any of the following support mechanisms: (1) CJRS, (2) SEISS, (3) BRH, (4) VATD and (5) TtP.

We also include several controls. We base our selection of control variables on those used in the wider literature, incorporating the same controls as in Clampit et al. (2022). Thus, we control for size with a logarithmic size variable that captures the number of employees in 2020. We include a dummy variable that equals 1 when SMEs are in transport, retail and food service/accommodation, as businesses in these sectors were more affected by COVID-19 constraints, such as social distancing in the service sector (Belitski et al., 2022) and were explicitly targeted by such government support instruments as BRH (Matikonis, 2020). We also include a dummy taking a value of 1 when SMEs are located in an urban area to control for differences in the operating environment and type of firms (Phillipson et al., 2019; Smallbone et al., 2003) as well as to accommodate differences in the spread of COVID-19 (Huang et al., 2021). Finally, our rich data enables us to control for the ethnic minority lead (EML) SMEs, which was identified as an important area to explore with future research in Clampit et al. (2022) and Martinez Dy and Jayawarna (2020), with a dummy taking a value of 1 for SMEs that are EML.

### Regression models

Given the ordinal structure of the dependent variables, ordinal regression is appropriate. Ordinal regression has been extensively applied in small business research (Kammerlander, 2016). To test for the presence of multicollinearity, we obtained the variance inflation factors (VIFs). This did not reveal any issues, with the highest VIF of 1.37. As a further robustness check, models are built in three steps. The initial models include only the control variables. The variables measuring DCs and government support are then added. The final model specifications include the control variables, DCs, government support and the interaction term between DCs and government support. At each stage of the model building process, we evaluated the improvement in residual deviance and Akaike information criterion (AIC). Further sensitivity analyses are reported in Appendix 2 by repeating the analyses with data from various time periods and excluding non-employing businesses.

## Analysis and results

We start by reporting descriptive statistics in Table 1. We see that in our sample most SMEs (64%, SD = 0.48) were located in urban areas, and just over one quarter (29%, SD = 0.45) operated in the transport, retail, food service, or accommodation industries. On average, SMEs in the sample have six employees (log mean = 1.71, SD = 1.45), indicating that the sample consists primarily of micro businesses. As reported in Appendix 1, the figures stay very similar once we further extend the collection period to March 2021.

**Table 1.** Descriptive statistics.

Variables	Count	Mean	SD
Change in employment			
2019–2020	1421	2.12	0.70
2020–2021	1294	2.14	0.56
Controls			
Urban dummy	1421	0.64	0.48
Sector dummy	1421	0.29	0.45
Ethnic minority lead dummy	1421	0.05	0.21
Size (log)	1421	1.71	1.45
Study variables			
Dynamic capabilities	1421	1.22	1.05
Government support	1421	1.18	1.10
Other variables			
CJRS	1421	0.51	0.50
SEISS	1421	0.11	0.31
BRH	1421	0.18	0.38
VAT deferrals	1421	0.29	0.45
Time to pay	1421	0.10	0.30

CJRS: Coronavirus Job Retention Scheme; SEISS: Self-Employment Income Support Scheme; BRH: Business Rates Holiday.

On average SMEs develop 1.22 DCs. However, this varies substantially across firms, as indicated by a standard deviation of 1.05. The SMEs received support from an average of 1.18 COVID-19-related schemes, but this variable also has a high standard deviation of 1.1, highlighting the variability in receipt of support. We also report separate statistics for each scheme. Around 51% (SD = 0.5) claimed CJRS, with a further 11% (SD = 0.31) claiming SEISS. As expected, based on the lower expenditure, other assistance initiatives were less widespread, with 29% (SD = 0.45) using VATDs, 18% (SD = 0.38) BRH and only 10% (SD = 0.3) HMRC’s TtP.

Table 2 supplements the descriptive statistics with the Spearman rank-order correlations, which show the magnitude and direction of association between two ordinal or numerical variables. We find a small positive correlation (*ρ* = 0.13) between DCs and employment growth aspirations after the early stages of the COVID-19 pandemic. The relationship between government assistance and DCs is also positive but small (*ρ* = 0.22). Small positive correlations between government assistance and employment changes during the first lockdown (*ρ* = 0.1) and beyond (*ρ* = 0.06) can also be observed.

**Table 2.** Spearman rank-order correlation coefficients.

Variables	ΔEmp 19–20	ΔEmp 20–21	DCs	Gov. sup.	Size (log)
ΔEmp 2019–2020	1.00				
ΔEmp 2020–2021	0.03	1.00			
DCs	0.04	0.13	1.00		
Government support	0.10	0.06	0.22	1.00	
Size (log)	−0.02	0.08	0.22	0.48	1.00

DCs: dynamic capabilities.

Table 3 presents the results of the ordinal regression models for changes in employment during and immediately after the first lockdown (models 1, 2 and 3) and employment growth aspirations after that (models 4, 5 and 6). For each dependent variable, we first present the regression models showing the control variables (models 1 and 4). The independent variables of DCs and government support are then added in models 2 and 5. The full models (models 3 and 6) then also include the interaction term between DCs and government support to test for a moderation effect. Table 3 also reports the residual deviance and AIC. Lower residual deviance in the final models indicates that the models with interaction terms predict the outcome variable better than alternative specifications. Similarly, lower AIC values in the full models indicate a better overall fit.

**Table 3.** Ordinal regression results for SMEs.

Dep. variable	Δ Employment 2019–2020				Δ Employment 2020–2021			
Notation	model 1 *b*_1_ (SE)	model 2 *b*_2_ (SE)	model 3 *b*_3_ (SE)	Exp (*b*_3_)	model 4 *b*_4_ (SE)	model 5 *b*_5_ (SE)	model 6 *b*_6_ (SE)	Exp (*b*_6_)
Study variables								
Dynamic capabilities		0.02 (0.05)	0.01 (0.07)	1.01		0.24*** (0.06)	0.44*** (0.09)	1.56
Government support		0.32*** (0.06)	0.32*** (0.08)	1.37		−0.04 (0.07)	0.20** (0.10)	1.22
Moderating effects			0.005 (0.04)	1.004			−0.17*** (0.05)	0.84
Controls								
Urban dummy	−0.05 (0.11)	−0.005 (0.11)	−0.005 (0.11)	0.995	−0.05 (0.12)	−0.08 (0.12)	−0.10 (0.12)	0.91
Sector dummy	0.36*** (0.12)	0.20* (0.12)	0.20* (0.12)	1.23	0.18 (0.13)	0.11 (0.14)	0.13 (0.14)	1.14
Ethnic minority lead dummy	0.19 (0.25)	0.11 (0.25)	0.11 (0.25)	1.11	0.28 (0.28)	0.24 (0.28)	0.26 (0.28)	1.3
Size (log)	−0.02 (0.04)	−0.13*** (0.04)	−0.13*** (0.04)	0.88	0.11*** (0.04)	0.09* (0.05)	0.08* (0.05)	1.09
Intercepts								
Decrease | Stayed the same	−1.44*** (0.11)	−1.27*** (0.12)	−1.28*** (0.13)		−2.08*** (0.14)	−1.93*** (0.15)	−1.72*** (0.16)	
Stayed the same | Increased	0.86*** (0.11)	1.07*** (0.11)	1.06*** (0.13)		1.38*** (0.13)	1.56*** (0.14)	1.8*** (0.16)	
Tests								
Residual dev.	2891	2856	2856		2144	2128	2117	
AIC	2903	2872	2874		2156	2144	2135	

The measures are reported on the logistic scale. The odds of coefficients for models 3 and 6 are also provided to aid interpretability.

AIC: Akaike information criterion.

* *p* < 0.1. **p < 0.05. ***p < 0.01.

Focusing first on the control variables, the regression results of model 3 indicate that if SMEs operate in the transport, retail and food service/accommodation sectors, the odds of increasing or sustaining employment (vs decreasing) increase by 23% (*b*_3_ = 0.20, SE = 0.12), holding all other variables constant. However, this relationship was not sustained after the early stages of the pandemic, as revealed by the lack of significant relationships with the dependent variable of future growth aspirations in model 6. Whilst the business size is significantly negatively (*b*_3_ = −0.13, SE = 0.04) related to employment change during and immediately after the first lockdown, but it is found to be significantly (at 10% confidence level) positively (*b*_6_ = 0.08, SE = 0.05) related to future growth. These relationships are broadly sustained in other specifications, reported in Appendix 2. Furthermore, models 8 and 10 (in Table A2, Appendix 2) that consist of larger sample sizes also capture significant positive relationships in terms of the urban area and EML dummies.

### Test of hypotheses

To formally test our hypotheses, we focus on the results from the full models (models 3 and 6), as reported in Table 3. *Hypothesis 1* posits a significant positive relationship between DCs and employment changes after the initial stages of COVID-19 directly after the first lockdown. During the early stages of COVID-19, the estimated DCs’ coefficient is very close to 0 and insignificant (*b*_3_ = 0.01, *p* = 0.89; 95% confidence interval (CI) = −0.13–0.15), but the effect becomes positive and highly statistically significant (*b*_6_ = 0.44, *p* < 0.01; 95% CI = 0.27–0.62) when looking further into the future at expected employment growth. The odds of increasing or sustaining employment (vs decreasing) in the future increase by 56% with each DC, holding constant all other variables. Our results, therefore, support *Hypothesis 1* and point to the positive effect of DCs on employment growth aspirations.

*Hypotheses 2* focuses on the effectiveness of government assistance. Specifically, we posit that acquiring government assistance positively contributes to SME employment during the early stages of the COVID-19 pandemic following the first lockdown. In support of *Hypothesis 2*, the coefficient of the government support is highly significant and positive (*b*_3_ = 0.32, *p* < 0.01; 95% CI = 0.16–0.48), suggesting that the odds of increasing or sustaining employment (vs decreasing) increase 37% with each COVID-19-related scheme that SMEs receive funds from, holding constant all other variables. It is also worth noting that similar, only slightly less uniform, as indicated by the wider confidence interval, relationships are sustained after the early stages of the COVID-19 pandemic (*b*_6_ = 0.2, *p* < 0.05; 95% CI = 0.09–0.39).

Finally, to test *Hypothesis 3*, which posits moderating effect, we estimate the interaction term coefficient between the number of government support programmes and DCs. In support of *Hypothesis 3*, we find a negatively significant coefficient of −0.17 (*p* < 0.01, 95% CI = −0.27 to −0.07) with a dependent variable of future employment growth aspirations.

### Sensitivity checks

We conduct sensitivity checks and confirm that these relationships hold to a large extent also for other COVID-19 periods. In Table A2 (Appendix 2), we start by extending the collection period to include the second lockdown with models 7 and 9. The estimated DCs coefficient for future employment growth aspirations is slightly lower but still positive and statistically significant (*b*_9_ = 0.3, *p* < 0.01; 95% CI = −0.2–0.39), supporting *Hypothesis 1.* In support of *Hypotheses 2 and 3*, we also find a significant positive coefficient for government support after the second COVID-19 lockdown (*b*_7_ = 0.30, *p* < 0.01; 95% CI = 0.21–0.39) and a negatively significant coefficient for moderating interaction for future employment growth (*b*_9_ = −0.11, *p* < 0.01, 95% CI = −0.17 to −0.05). Similar trends are observed once we extend the collection to March 2021 (models 8 and 10 in Table 5, Appendix 2), capturing most of the restrictions associated with COVID-19. *Hypotheses 1, 2 and 3* are supported with *b*_10_ of 0.29 (*p* < 0.01; 95% CI = 0.22–0.36), *b*_8_ of 0.24 (*p* < 0.01; 95% CI = 0.17–0.31) and *b*_10_ of −0.08 (*p* < 0.01; 95% CI = 0.09–0.24), respectively.

We further conduct sensitivity checks with data limited only to SMEs with employees in Table A3 (Appendix 2). The estimated moderating effect is now even more negative and still highly significant (*b*_12_ = −0.2, *p* < 0.01, 95% CI = −0.31 to −0.08) after the early stages of COVID-19, providing evidence supporting *Hypothesis 3*. In support of *Hypotheses 1 and 2*, we also find a significant positive coefficient for government support in the early stages of COVID-19 (*b*_11_ = 0.34, *p* < 0.01; 95% CI = 0.16–0.51) and a significant positive coefficient for DCs after the early stages of COVID-19 (*b*_12_ = 0.51, *p* < 0.01, 95% CI = 0.3–0.72).

## Discussion

### Dynamic capabilities

The COVID-19 pandemic forced SMEs to adapt, drawing on their DCs and government support mechanisms. Despite this observation, a gap remains in the literature regarding the role of DCs and government support in SME employment, to which this article contributes. Our findings highlight the positive effects of DCs in facilitating employment growth aspirations as society moves out of the pandemic. This finding supports the work of Clampit et al. (2022) and Dejardin et al. (2022), who find that DCs help SMEs recover from the pandemic. However, this past literature did not focus specifically on employment, which is a particularly important factor during the COVID-19 pandemic, with substantial government resources devoted to securing jobs. The importance of DCs in enabling SMEs to adapt to changes in the external environment during crises has also been identified in other contexts, such as the 2008 financial crisis (Makkonen et al., 2014), earthquakes (Battisti and Deakins, 2017; Mahto et al., 2022) and war (Mansour et al., 2019). We join these studies in their efforts to highlight the importance of DCs in enabling SMEs to cope with external shocks.

Our research extends existing knowledge by addressing the previous discord (Baia et al., 2020; Wilden et al., 2016) on how DCs affect the performance of SMEs. We find evidence that the initial rationale offered by Teece et al. (1997) is valid in the context of COVID-19. DCs are critical in sensing and seizing opportunities and the reconfiguration of resources to enable the adaptation of business models (Teece, 2018), which is particularly important during times of high disruption such as the COVID-19 pandemic (Dyduch et al., 2021; Ozanne et al., 2022) when conditions are dynamic (Helfat and Peteraf, 2009; Schilke, 2014; Teece et al., 1997). This means that SMEs with DCs can better adapt to shocks by taking advantage of opportunities, leading to increased employment growth aspirations.

Our findings demonstrating the positive effect of DCs on employment supplement the wider literature indicating that DCs are positively associated with a range of performance measures during the COVID-19 pandemic, including greater operation levels and turnover (Clampit et al., 2022; Dejardin et al., 2022), sales volume growth, sales growth, profit, return on investment, employee productivity, and market share (Li et al., 2022), competitive advantage (Fainshmidt et al., 2016) and other industry-specific outcomes (Marco-Lajara et al., 2021). We extend these studies to employment growth and further show that SMEs with greater operational levels and turnover (Clampit et al., 2022; Dejardin et al., 2022) resulting from DCs utilised during the first lockdown had more confidence that they could adapt and grow in employment despite the impact of the pandemic. Conversely, firms that do not possess DCs may be unable to adapt to shocks, leading to reduced employment. Ultimately our results indicate that SMEs that do not draw on DCs during the pandemic have poorer outcomes in terms of employment growth aspirations. Our findings are thus also supplementary to the wider literature, which indicates that a failure to draw on DCs during the COVID-19 pandemic decreases financial performance (Clampit et al., 2022) as well as increasing the chances of failure (Ozanne et al., 2022).

We also contribute to the existing knowledge by shedding light on the conflicting evidence (Barreto, 2010) around the possible mechanisms through which DCs operate. We find nuanced relationships in terms of the timing of the effects of DCs on employment. We thus echo others (Andersen et al., 2022; Pavlou and Sawy, 2010; Wilhelm et al., 2015) and extend their logic on the importance of pace during crises. We find that SMEs that utilised their DCs to implement changes in their operations during the first lockdown, are likely to have higher subsequent employment growth than those that employed this strategy later in the pandemic, as indicated by lowering DC coefficients (*b*_6_ > *b*_9_ > *b*_10_) in Table A3 (Appendix 2). We thus, also contribute to disentangling one of the main managerial dilemmas of timing versus sustainability (Andersen et al., 2022). Our findings indicate that SMEs that promptly use their DCs to adapt their structures and strategies to leverage opportunities during a crisis are likely to be more successful in terms of longer-term employment growth than those that opt for a wait-and-see approach and implement changes later in a crisis.

### DCs and government support

The findings show an overall positive impact of government support on employment during the first lockdown of the COVID-19 pandemic. These findings thus contribute to the insights from other studies that focused on individual support schemes (Bartik et al., 2020b; Calabrese et al., 2022; Cowling and Dvouletý, 2022; Cowling et al., 2022; Doniger and Kay, 2021) by capturing the impact of the quantity of support schemes that SMEs received on employment change. We also see that the support, at least to some extent, benefited the economy by sustaining and creating employment, as such policies typically intend to do (Cowling and Dvouletý, 2022; Kersten et al., 2017). Our study thus highlights the crucial role that government support mechanisms have in protecting SME employment during shocks.

In addition to the direct impact of DCs and government support on SME employment, we also go further than existing studies focusing on DCs (Clampit et al., 2022; Cowling et al., 2022; Dejardin et al., 2022; Yue and Cowling, 2021) by examining the impact of government support alongside DCs, providing evidence on how DCs are affected by government assistance during a crisis. The findings show that the impact of government support in discouraging SMEs from effectively using DCs varies through different stages of the pandemic. We find that substantial support during the pandemic moderates the relationship between DCs and growth aspirations in the later stages and after the pandemic.

This negative moderating effect indicates more nuanced relationships. We find evidence in support of the reasoning that generous support initiatives also could have sustained and created new, less viable firms (Acharya et al., 2020; McGowan et al., 2018) that could potentially take a share of the market from SMEs with DCs, contributing to the lack of overall effectiveness of DCs and subsequent lower employment growth. Similar deficiencies are likely to be present because of the design of COVID-19 support instruments that, in some cases, were argued (Matikonis, 2020, 2022) to be distortive and suffer from mistargeting. For SMEs that receive funds from several COVID-19 schemes, the negative relationship could also arise because of opportunity costs associated with a shift towards developing political management capabilities (Oliver and Holzinger, 2008) through the increasingly dynamic political environment with changing support mechanisms at the expense of developing higher quality DCs that could benefit longer-term employment growth.

On the other hand, SMEs with higher levels of DCs seem to receive less benefit from government support in terms of future employment growth, possibly because they are already more resilient to shocks (Dovbischuk, 2022; Ozanne et al., 2022). Some firms might also choose to rely on government support rather than drawing on their DCs, in effect wasting any opportunity to learn from adapting to the new market conditions. DCs are known to be developed through experience (Bingham et al., 2015), so this could have a negative impact on these SMEs in the long term as they may be unable to catch up with competitors who successfully adapted to the pandemic, of which we already have numerous examples (Davidsson et al., 2021; Guckenbiehl and Corral de Zubielqui, 2022; Klyver and Nielsen, 2021). The findings also suggest that government support is more critical in maintaining or increasing employment levels in SMEs that are unable to draw on their own DCs to adapt to the pandemic.

### Implications for managers and policymakers

Our findings have implications for managers and policymakers. The findings direct to strategies that the former could apply depending on the resources available to SMEs. Attaining all available government support could be most beneficial in instances where SMEs do not have sufficient resources to cope with crises themselves and are likely to shrink or close. Immediately once the support is received, managers need to evaluate and consider how to create, extend or modify their resource base, keeping in mind that over-relying on this short-term support may not sustain competitive advantage in the longer term. SMEs that can survive should not spend extensive time and resources researching and applying for government assistance programmes but favour moving beyond tactical adaptions to the development of DCs that will enable resilience towards external shocks (Ali et al., 2022).

Our findings also provides evidence for policymakers regarding the longer-term effects of expansive support programmes during a crisis. Although government assistance contributes to reducing short-term unemployment during COVID-19, the support instruments also distort the overall efficiency of DCs and, in this way, contribute to lowering employment growth in the longer term. From the results presented above, and in line with the previous criticism of other support programmes (Matikonis, 2020), this differential impact of government support mechanisms highlights the importance of targeting interventions towards the firms most likely to benefit. More effort should also be made by policymakers to tackle the complexity of the increasingly expansive relief packages that are disjoint, quickly changing and challenging to navigate. This could potentially reduce the unintended negative consequences of the support.

## Conclusions

This article calls attention to the role of government policy intervention in discouraging SMEs from effectively exercising DCs during a crisis. We observe the positive impact of government support on employment in the early stages of the COVID-19 pandemic, contributing to the existing literature that investigated individual support schemes (Clampit et al., 2022; Cowling et al., 2022; Dejardin et al., 2022; Yue and Cowling, 2021). We also find that DCs positively affect employment after the early stages of the COVID-19 pandemic, further supplementing insights from previous studies that used other performance indicators (Clampit et al., 2022; Dejardin et al., 2022; Fainshmidt et al., 2016; Marco-Lajara et al., 2021). Most importantly, we find that government support negatively moderates the link between DCs and employment growth aspirations, further extending the DC theory. We suggest that this relationship is likely to be an effect of poorly designed COVID-19 support instruments subsidising less viable SMEs and, in this way, contributing to the shift away from effectively employing DCs towards alternatives, including political management capabilities.

### Limitations and future research

Future studies could empirically explore the avenues for this negative relationship. A more qualitative study could better inform us of the reasons behind the negative longer-term impact of government assistance on the effectiveness of DCs. The wider literature also highlights a lack of understanding of the mechanisms by which DCs lead to performance outcomes during the pandemic (Ozanne et al., 2022). Although we contribute to this understanding, future studies could consider the role of additional constructions, such as SME size differentials and more detailed time effects, alongside government support.

Although the large-scale microdata brought many benefits to our analysis, including representative sample size, the usage of this secondary quantitative data also has inherent limitations. Firstly, our dependent variable covers employment changes between September/October 2020 and 12 months prior, including some employment movement before the COVID-19 pandemic. However, this limitation, to some extent, is lessened once we consider that looking at a change over 12 months allows us to control for seasonal changes in employment. The magnitude of COVID-19 and its associated restrictions are also likely to be the primary driver of employment change in that period, which we also observe through sensitivity analyses in Appendix 2 when adjustments to the data collection period do not result in substantial changes in relationships. Whilst another dependent variable focuses on employment growth aspirations.

Even though measures of growth aspirations are commonly applied in the wider entrepreneurship literature (Darnihamedani and Terjesen, 2022; Davis and Shaver, 2012; Efendic et al., 2015), it suffers from relying on the projection, which future studies could test. The cross-sectional survey design also has its standard limitations, including measurement, causality and respondents’ interpretations of survey questions, which we at least partly mitigate through extensive sensitivity analyses, including various specifications and samples, as reported in Appendix 2.

The ideal data would precisely capture historical monetary and non-monetary contributions towards the development of particular DCs up to COVID-19, in the notion of Helfat et al. (2007), to what extent and at what pace these then trigged the change during COVID-19 and how this change affected their outcomes during and post-COVID-19. More realistically, our study could be supplemented by a similar analysis of purpose-made surveys during other crises, with alternative conceptualisations of the DCs, for instance, through the adoption of a more gradual scale when estimating DCs, as explained by Teece et al. (2016), and data collections at different timeframes.

## Appendix 1

**Table A1.** Descriptive statistics – full data.

Variables	Count	Mean	SD
Change in employment			
2019–2020	7625	2.12	0.72
2020–2021	7152	2.19	0.55
Controls			
Urban dummy	7625	0.65	0.48
Sector dummy	7625	0.27	0.45
Ethnic minority lead dummy	7625	0.05	0.21
Size (log)	7625	1.82	1.52
Study variables			
Dynamic capabilities	7625	1.22	1.05
Government support	7625	1.17	1.08
Other variables			
CJRS	7625	0.53	0.5
SEISS	7625	0.12	0.32
BRH	7625	0.17	0.37
VAT deferrals	7625	0.27	0.44
Time to pay	7625	0.09	0.29

CJRS: Coronavirus Job Retention Scheme; SEISS: Self-Employment Income Support Scheme; BRH: Business Rates Holiday.

## Appendix 2

**Table A2.** Ordinal regression results for small and medium-sized enterprises with data limited to various collection periods.

Dep. variable	Δ Employment 2019–2020			Δ Employment 2020–2021		
Notation	Model 3 *b*_3_ (SE)	Model 7 *b*_7_ (SE)	Model 8 *b*_8_ (SE)	Model 6 *b*_6_ (SE)	Model 9 *b*_9_ (SE)	Model 10 *b*_10_ (SE)
Study variables						
Dynamic capabilities	0.01 (0.07)	0.01 (0.04)	−0.03 (0.03)	0.44*** (0.09)	0.30*** (0.05)	0.29*** (0.04)
Government support	0.32*** (0.08)	0.30*** (0.04)	0.24*** (0.03)	0.20** (0.10)	0.18*** (0.05)	0.17*** (0.04)
Moderating effects	0.005 (0.04)	0.01 (0.02)	0.05** (0.02)	−0.17*** (0.05)	−0.11*** (0.03)	−0.08*** (0.02)
Controls						
Urban dummy	−0.005 (0.11)	0.06 (0.06)	0.11** (0.05)	−0.10 (0.12)	−0.04 (0.07)	−0.002 (0.05)
Sector dummy	0.20* (0.12)	0.17** (0.07)	0.13*** (0.05)	0.13 (0.14)	0.04 (0.08)	−0.03 (0.06)
Ethnic minority lead dummy	0.11 (0.25)	0.18 (0.14)	0.28*** (0.11)	0.26 (0.28)	0.39** (0.17)	0.30** (0.13)
Size (log)	−0.13*** (0.04)	−0.08*** (0.02)	−0.07*** (0.02)	0.08* (0.05)	0.13*** (0.02)	0.18*** (0.02)
Intercepts						
Decrease | Stayed the same	−1.28*** (0.13)	−1.14*** (0.08)	−1.07*** (0.06)	−1.72*** (0.16)	−1.92*** (0.09)	−1.93*** (0.07)
Stayed the same | Increased	1.06*** (0.13)	1.06*** (0.07)	1.04*** (0.06)	1.8*** (0.16)	1.75*** (0.09)	1.8*** (0.07)
Tests						
Residual dev.	2856	9214	15,734	2117	6695	11,304
AIC	2874	9232	15,752	2135	6713	11,322
Observations						
	1421	4507	7625	1295	4211	7156

The measures are reported on the logistic scale. Models 3 and 5 are similar to those reported in Table 3 and include the collection period between September and October 2020. Models 7 and 9 further extend it to December 2020, and models 8 and 10 to all data (September 2020 to March 2021).

AIC: Akaike information criterion.

* *p* < 0.1. ***p* < 0.05. ****p* < 0.01.

**Table A3.** Ordinal regressions including non-employing small and medium-sized enterprises.

Dep. variable	Δ Employment 2019–2020		Δ Employment 2020–2021	
Notation	Model 3 *b*_3_ (SE)	Model 11 *b*_11_ (SE)	Model 6 *b*_6_ (SE)	Model 12 *b*_12_ (SE)
Study variables				
Dynamic capabilities	0.01 (0.07)	−0.03 (0.09)	0.44*** (0.09)	0.51*** (0.11)
Government support	0.32*** (0.08)	0.34*** (0.09)	0.20** (0.10)	0.24** (0.10)
Moderating effects	0.005 (0.04)	−0.001 (0.05)	−0.17*** (0.05)	−0.20*** (0.06)
Controls				
Urban dummy	−0.005 (0.11)	−0.03 (0.12)	−0.10 (0.12)	−0.05 (0.14)
Sector dummy	0.20* (0.12)	0.20 (0.13)	0.13 (0.14)	0.10 (0.15)
Ethnic minority lead dummy	0.11 (0.25)	0.03 (0.28)	0.26 (0.28)	0.25 (0.32)
Size (log)	−0.13*** (0.04)	−0.02 (0.05)	0.08* (0.05)	0.09 (0.06)
Intercepts				
Decrease | Stayed the same	−1.28*** (0.13)	−0.63*** (0.19)	−1.72*** (0.16)	−1.12*** (0.22)
Stayed the same | Increased	1.06*** (0.13)	1.08*** (0.19)	1.8*** (0.16)	1.75*** (0.22)
Tests				
Residual dev.	2856	2179	2117	1709
AIC	2874	2197	2135	1727
Observations				
	1421	1025	1295	925

The measures are reported on the logistic scale. Models 3 and 6 are similar to those reported in Table 3 and include all SMEs. Models 11 and 12 exclude non-employing businesses.

AIC: Akaike information criterion.

* *p* < 0.1. ***p* < 0.05. ****p* < 0.01.

## Notes

1. For instance, supermarkets and other food retailers were required to adhere to social distancing measures, including the severe regulation of footfall and restaurant services were limited to only home delivery or collections (Barber et al., 2022; Brown and Kirk-Wade, 2021).

2. These and other support initiatives are now estimated to cost over £147 billion, an increase of 61% from the original estimates at the point of announcements (National Audit Office, 2022).

3. This was done to ensure that the periods of observation do not overlap. Fieldwork for the 2020 survey was conducted between September 2020 and April 2021. The complete 2020 sample consists of responses from 7636 SMEs. Due to missing data, the sample was reduced to 7625 usable responses for the sensitivity analysis focusing on employment change between 2019 and 2020 and 7156 on employment change between 2020 and 2021 that are reported in Appendix 2.
